# Improvement of heat stress tolerance in soybean (*Glycine max* L), by using conventional and molecular tools

**DOI:** 10.3389/fpls.2022.993189

**Published:** 2022-09-26

**Authors:** Guan Jianing, Gai Yuhong, Guan Yijun, Adnan Rasheed, Zhao Qian, Xie Zhiming, Athar Mahmood, Zhang Shuheng, Zhang Zhuo, Zhao Zhuo, Wang Xiaoxue, Wei Jian

**Affiliations:** ^1^Rice Research Institute, Shenyang Agricultural University, Shenyang, China; ^2^College of Agronomy, Jilin Agricultural University, Changchun, China; ^3^College of Life Sciences, Northwest A&F University, Yangling, China; ^4^College of Life Sciences, Changchun Normal University, Changchun, China; ^5^College of Life Sciences, Baicheng Normal University, Baicheng, China; ^6^Department of Agronomy, University of Agriculture Faisalabad, Faisalabad, Pakistan; ^7^College of Life Sciences, Jilin Normal University, Changchun, China

**Keywords:** soybean, heat stress, yield, molecular techniques, global warming

## Abstract

The soybean is a significant legume crop, providing several vital dietary components. Extreme heat stress negatively affects soybean yield and quality, especially at the germination stage. Continuous change in climatic conditions is threatening the global food supply and food security. Therefore, it is a critical need of time to develop heat-tolerant soybean genotypes. Different molecular techniques have been developed to improve heat stress tolerance in soybean, but until now complete genetic mechanism of soybean is not fully understood. Various molecular methods, like quantitative trait loci (QTL) mapping, genetic engineering, transcription factors (TFs), transcriptome, and clustered regularly interspaced short palindromic repeats (CRISPR), are employed to incorporate heat tolerance in soybean under the extreme conditions of heat stress. These molecular techniques have significantly improved heat stress tolerance in soybean. Besides this, we can also use specific classical breeding approaches and different hormones to reduce the harmful consequences of heat waves on soybean. In future, integrated use of these molecular tools would bring significant results in developing heat tolerance in soybean. In the current review, we have presented a detailed overview of the improvement of heat tolerance in soybean and highlighted future prospective. Further studies are required to investigate different genetic factors governing the heat stress response in soybean. This information would be helpful for future studies focusing on improving heat tolerance in soybean.

## Introduction

**FIGURE 1:**
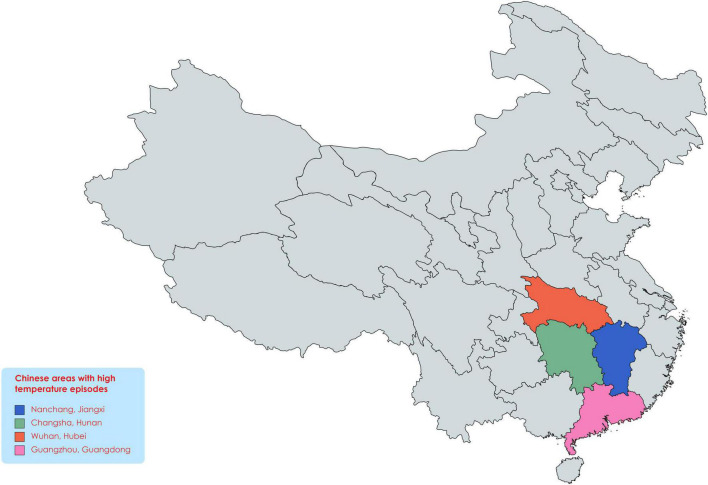
Areas with extreme heatwaves in China. As global warming in rising, the heat waves in these areas continue to increase, which will have devastating effects on crop production in the coming time.

Abiotic stresses are severe environmental factors affecting crop growth and yield (Yang et al., 2017; Saleem et al., 2020; Zafar-Ul-Hye et al., 2020), undermining global food security (Rasheed et al., 2020a,b,c, 2021a,b), and affected the large area under cultivation (Ashfaq et al., 2021). Heat stress is most disturbing abiotic factor that disturbs plant growth, development, and yield (Mittler and Blumwald, 2010; Suzuki et al., 2014; Nakagawa et al., 2020). For example, extreme heat stress occurred in the United States of America (USA), resulting in a yield loss estimated about 7.1 billion US dollars (Mittler, 2006). Comparable events happening worldwide and caused instability in social and economic aspects and mass migration, and China is also facing extreme heat waves (Figure 1) in many regions (Ault, 2020; Xie et al., 2021). Multiple research studies have shown the effects of heat stress on soybean growth, yield, and quality (Cohen et al., 2021b). In soybean, efforts have been made to develop genotypes with enhanced yield under extreme and prolonged heat waves (Mittler, 2006). Many studies have observed yield loss when soybean was exposed to short-term and long-term heat waves (Hatfield and Dold, 2019; Thomey et al., 2019; Zandalinas et al., 2020) and season-long warming (Ruiz-Vera et al., 2013). Soybean meal is used as the main source of protein in livestock feeding (Consultation, 2004) and harvested on 82 million acres in the USA in 2020 (Ortiz et al., 2022).

Heat stress significantly affected the soybean seed germination, resulting in poor seed germination (Hamayun et al., 2021; Veas et al., 2021), increased seed vulnerability to pathogen attack, and reduced its economic value (Wang et al., 2018). Climate change is expected to be more severe and increase the severity of heat waves and their impact on seed development during the current century (Chebrolu et al., 2016; Huang et al., 2019). Heat stress affects soybean root growth, alters morphology, and restricts elongation (de Moraes and Gusmao, 2021). Heat stress reduces carbon assimilation, causes stomatal closure and membrane injury, and decreases the activity of several enzymes (Farooq et al., 2009). Heat stress impairs the metabolic pathways (Kosová et al., 2011), which results in altered plant growth (Kosová et al., 2011; Liu et al., 2014). Climate change is expected to increase the incidence and severity of summer heat waves (Balfagón et al., 2022; Ullah et al., 2022), and the impact of heat stress on seed development will become more widespread (Haider et al., 2022) and prolonged during current century (Chebrolu et al., 2016; Sun et al., 2022). Episodes of heat waves are expected to increase in intensity in the coming time due to global warming and climate changes (Balfagón et al., 2019; Ault, 2020; Grossiord et al., 2020). Hence, breeding crops for increased tolerance to heat stress is one of the most critical needs of modern agriculture (Bailey-Serres et al., 2019; Zandalinas et al., 2020). In addition to its effects on growth and biomass, its prolonged period could have a more deadly impact on yield and quality (Cohen et al., 2021b).

Therefore, the presence of heat-tolerant soybean genotypes is vital for global food security, and exploiting genetic variation for heat response could play a significant role in crop development (Ergo et al., 2021; Rustgi et al., 2021). Quantitative trait loci (QTL) mapping is a most powerful technique (Xu et al., 2013; Zhang et al., 2017; Rasheed et al., 2022b), which allows the identification of genomic regions controlling heat tolerance in soybean (Bazzer and Purcell, 2020; Gillman et al., 2021). Soybean plants have been exposed to extreme heat waves, and many significant QTL have been discovered. Soybean exposure to heat waves under controlled conditions would identify novel QTL, which can be cloned and transferred to develop heat-tolerant cultivars. Likewise, genetic engineering plays a crucial role in enhancing heat tolerance in soybean (Rahman et al., 2022; Vital et al., 2022). These techniques have brought fruitful results in molecular biology research (Xu et al., 2019). Likewise, the transcriptome technique has discovered several genes governing the heat tolerance mechanism in soybean (Wang et al., 2018; Katam et al., 2020). CRISPR/Cas9, a novel gene-editing tool, can potentially edit any desired gene of interest in the soybean genome to bring novel variation for heat tolerance (Duan et al., 2021). However, the large-scale application of this tool in soybean needs more studies and research. In the future, using different Cas9 variants would enable any desired mutation in the soybean genome (Kim, 2017; Huang et al., 2019).

Several studies deal with the improvement of soybean heat tolerance; however, complete genetic control of this trait is yet not fully understood. Heat waves are becoming more and more intense every year, creating a significant threat to global food security by reducing crop yields. This review has critically elaborated several classical and molecular techniques to develop heat-tolerant soybean genotypes. The current review highlighted the potential research gaps that should be covered in future research studies. This review would be a potential source of literature for future research studies dealing with heat tolerance in soybean.

## Effects of heat stress on soybean growth, physiological and biochemical parameters

**FIGURE 2:**
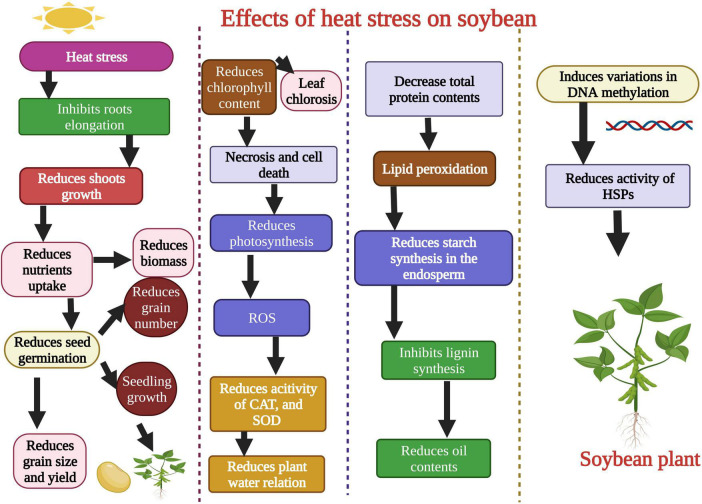
Effects of heat stress on morphological, physiological, biochemical, and molecular level of soybean. Heat stress affects the shoot growth, root growth, reduces seed germination, produces reactive oxygen species (ROS), decreases the activity of antioxidant enzymes, and total protein contents. Heat stress causes changes in DNA methylation and gene’s function. This Figure is created with BioRender.com.

Plants face series of harsh environmental factors including biotic and abiotic stresses (Ahmad et al., 2022; Khan et al., 2022). Heat stress negatively affects soybean growth and development. Chebrolu et al. (2016) evaluated soybean heat tolerant and susceptible genotypes under heat stress conditions. Heat stress (42°C) significantly affected the seeds germination (Figure 2) of the heat susceptible genotype, while these parameters remained unaffected in the heat-tolerant genotype. Heat stress affected the seed protein contents and protein storage vacuoles of soybean when plants were exposed to 42°C. Heat stress disturbs the vacuole structure and membrane integrity that stores protein (Krishnan et al., 2020). Likewise, in another study, it was noted that extreme heat stress decreased the contents of salicylic acid (SA) in soybean after exposure for 5–10 days (Khan et al., 2020). Heat stress also affected the activity of different metabolites and amino acids in soybean. Carbohydrates, lipids, proteins, and secondary metabolites were involved under heat stress, and their concentration was reduced when plants were exposed to extreme heat stress (Das et al., 2017). Heat stress negatively affected the seed production in soybean genotypes, ultimately affecting yield. Cohen et al. (2021a) evaluated the soybean genotypes (Magellan and PI 548313) under heat waves to study seed production and yield per plant. Results revealed that PI 548313 yielded more seeds than Magellan under heat stress. These findings indicated that heat stress could affect any plant growth stage and decrease the final yield per plant in soybean (Cohen et al., 2021a). Heat stress damaged the plant leaf and reduced soybean soluble sugar and proline contents when plants were exposed to heat stress for 24 h (Wang et al., 2018).

Heat stress affects the soybean root growth and structure (Valdés-López et al., 2016). In another study, heat stress reduced soybean plant chlorophyll content (Figure 2). Heat stress also reduced the accessibility of assimilates to soybean grains and reduced the sink-grain metabolization from young leaves (Ergo et al., 2018). Sharifi et al. (2022) studied the detrimental effects of heat stress on soybean growth, physiology, and biochemical parameters. They found that heat stress reduced the shoot and dry root weight, induced oxidative damage, and decreased the stomatal conductance (Table 1; Sharifi et al., 2022). The soybean yield was also affected under heat stress conditions. High-intensity heat waves reduced the soybean yield by affecting flowering and pod filling stages (Thomey et al., 2019). In another study, Cohen et al. (2021a) studied the detrimental effects of heat stress on soybean reproductive organs like stigma, ovaries, pollens, and flowers, which resulted in lower seed production. Further studies are required for a detailed investigation of the effects of heat stress on the activity of antioxidants, osmolytes, and specific biochemical components of soybean. Heat-induced changes in the genetic material of soybean are critical to understanding to adopt preventive measures.

**TABLE 1:** Effects of heat stress on soybean.

Crop	Effects	References
Soybean	Heat stress-induced pollen sterility	Lindsey and Thomison, 2012
	Heat stress reduced fatty acid contents and altered the biochemical profile	Bellaloui et al., 2015
	Heat stress reduced seeds germination percentage and affected seedling growth	Chebrolu et al., 2016
	Heat stress reduced the root’s growth and altered their structure	Valdés-López et al., 2016
	Heat stress affected the seed coat wrinkling, coat boron, lignin, shattering, and hard seed	Bellaloui et al., 2017
	Heat stress reduced the concentration of lipids, metabolites, carbohydrates, and amino acids	Das et al., 2017
	Heat stress reduced the sink-grain metabolization from young leaves	Ergo et al., 2018
	Damaged leaf, reduced sugar and proline contents	Wang et al., 2018
	Heat stress affected flowering and pod filling stages and reduced yield	Thomey et al., 2019
	Heat stress reduced the antioxidant enzymes activity and epicuticular wax content	Jumrani and Bhatia, 2019
	Heat stress reduced the seed protein contents and disturbed the membrane integrity of seed-storing vacuoles	Krishnan et al., 2020
	Heat stress decreased the concentration of SA when plants were exposed to heat stress for 5 to 10 days	Khan et al., 2020
	Heat stress reduced the seed production and yield per plant	Cohen et al., 2021a
	Heat stress caused oxidative damage, lowered the net intercellular CO_2_, and stomatal conductance, and shoot dry weight	Sharifi et al., 2022
	Long-term heat stress reduced seed oil and protein concentration	Ortiz et al., 2022

## Molecular mechanism of heat tolerance in soybean

The molecular mechanism of heat tolerance is very complex and governed by multiple genes (Zhang et al., 2022a). Incomplete knowledge of molecular factors underlying heat tolerance in soybean has hindered the research progress (Zhang et al., 2022a). The plant adopts several techniques to cope with heat stress (Katam et al., 2020; Sharifi et al., 2022). Many genes, enzymes, and proteins are crucial in improving soybean heat stress tolerance. Heat stress induced the overexpression of the *DREB1* gene family, several dehydrins, and LEA genes, their over-representation between upregulated genes in soybean under heat stress (Kidokoro et al., 2015). Likewise, overexpression of enzymes involved in homeostasis, as well as accumulation of chaperone proteins, mainly heat shock proteins, were observed (Kosová et al., 2011).

Consequently, proteomic analysis under multiple stresses leads to a better understanding of the molecular paths and the molecules related to heat tolerance. Earlier studies also showed the relative abundance of leaf proteins under heat stress conditions in soybean (Ashoub et al., 2015). These studies showed that soybean plants adopt various pathways and mechanisms to cope with high-temperature circumstances. However, some recognized genes and pathways could be employed to improve heat tolerance in soybeans using molecular breeding approaches (Sabagh et al., 2020). Future studies on the molecular mechanism of heat stress tolerance in soybean would identify the different genes and pathways controlling heat tolerance in soybean.

## Classical breeding approaches to enhance heat tolerance in soybean

One way to meet future food demands is to develop heat-tolerant soybean cultivars that maintain high yields under heat waves. Heat tolerance is a polygenic trait. It is critical to identify the major contributors to this trait and use molecular markers for breeding using a specific breeding program (Rustgi et al., 2021). A successful breeding program needs significant genetic diversity for a given trait. Crossing closely related individuals can decline genetic variability (Gur et al., 2004). The primary step is the characterization of a shared gene pool, including landraces and wild relatives, which offers the identification of numerous tolerant genes for abiotic tolerance. Wild relatives are opportunistic resources for soybean breeding (Lam et al., 2010; Nawaz et al., 2018). Soybean wild relative *Glycine soja* is usually considered the uncultivated ancestor of the domesticated soybean (Stupar, 2010). *Glycine max* and *Glycine soja* are phenotypically dissimilar in many behaviors, but they readily cross with one another and give rise to productive hybrids for several key features (Stupar, 2010). Comparative genome sequencing of cultivated and wild soybean relatives will enhance our understanding of the limitations of domesticated germplasm and the potential to exploit wild germplasm for breeding programs (Stupar, 2010). The wild soybean genome has more allelic diversity than cultivated soybean. In an earlier study by Lam et al. (2010), resequencing 14 cultivated and 17 wild soybean genomes identified greater allelic diversity (Lam et al., 2010). These studies showed that wild soybean relatives are treasure troves to increase genetic diversity for abiotic stress tolerance by contributing novel alleles (Mammadov et al., 2018).

Significant efforts have been made using conventional breeding to screen heat-tolerant genotypes, which can be used to sustain growth and yield under changing environmental conditions (Jagadish et al., 2008).

**FIGURE 3:**
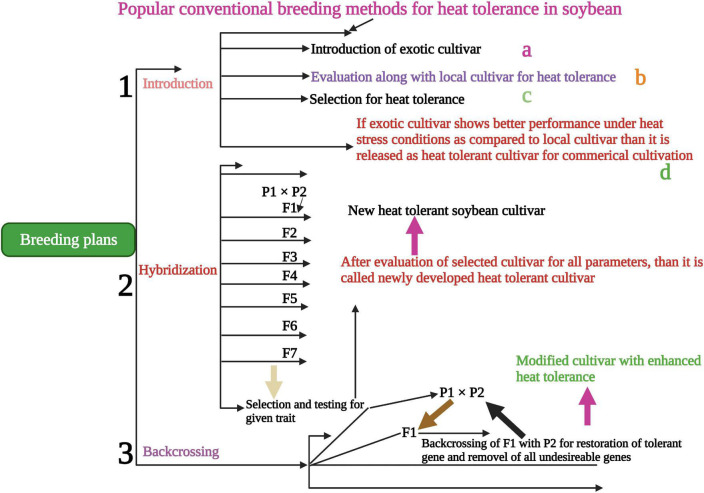
Conventional breeding methods for the development of heat-tolerant soybean cultivars. Hybridization, backcrossing and introduction are powerful traditional ways to develop tolerant soybean cultivars to sustain yield in heat stress conditions. This Figure is created with BioRender.com.

Conventional screening techniques for heat tolerance showed promising results in many crop species (Jagadish et al., 2008). Breeding programs were carried out in heat stress areas that endorse crop selection for thermo-tolerant traits. Understanding the genetic and physiological-based heat tolerance mechanisms will help to develop heat tolerant cultivars suitable for sustainable growth under heat waves (Shanmugavel et al., 2020). Alsajri et al. (2019) screened 64 soybean cultivars using certain morphological and physiological traits for heat stress tolerance. They have identified 45A46 (Table 2) high heat tolerant cultivar based on their results. Mass selection, hybridization (Figure 3), pure line selection, and backcrossing methods are widely used for breeding heat-tolerant soybean cultivars. Hybridization is one of the most reliable breeding methods and leads to the development of hundreds of heat-tolerant soybean cultivars (Singh and Nelson, 2015; Mammadov et al., 2018). The soybean line (TN09-239) is an MG5 line developed *via* four circles of backcrossing (Boehm et al., 2017), and the soybean line (PI587982A) is a plant introduction collected in Sichuan Sheng, China, and currently, USDA-GRIN germplasm collection. PI587982A has been confirmed to have prolonged tolerance to the adverse influences of heat stress during seed filling (Smith et al., 2008; Gillman et al., 2019). Modified breeding techniques would assist in speeding up the development of heat-tolerant soybean cultivars.

**TABLE 2:** List of heat-tolerant soybean varieties/lines.

Crop	Genotype	Cumulative high temperature response indices (CHTRI) and tolerance ranking
Soybean	45A46	26.28
	CZ 5375 RY	20.88
	CZ 4181 RY	24.17
	PI587982A	High
	TN09-239	High
	PI-471938	High
	SPS4 × 4RR	High
	GMLN012012017	High
	EC 538828	Medium

## Use of phytohormones for heat tolerance in soybean

Different conventional approaches have been used to enhance heat tolerance in soybean. Earlier reports suggested that certain plant hormones mitigated the toxic effects of heat waves in soybean. The plant has adopted various mechanisms that help to cope with increasing episodes of heat stress. These modifications include leaf orientation, changes in lipid composition in the membrane, activation of the antioxidant defense system, and hormonal regulation (Hasanuzzaman et al., 2013). Heat stress causes ROS overproduction and oxidative damage to DNA, proteins, and lipids (Liu et al., 2019; Haider et al., 2022). Phytohormones control plant growth and development under extreme heat waves. Mineral fertilizers greatly affect the crop growth and production (Amanullah Ilyas et al., 2021; Amjad et al., 2021), so eco-friendly techniques are required to enhance yield under abiotic stresses (Sabagh et al., 2019). A slight change in hormonal concentration affects the cellular dynamics; hence, they have a crucial role in controlling the plant response to heat stress (Ozga et al., 2017; Singh and Meena, 2020). In general, auxin (AX), gibberellins (GA), and cytokinin (CK) involve in the regulation of heat tolerance under heat waves (Ozga et al., 2017). Khan et al. (2020) revealed that heat stress increased the concentration of ABA and SA in soybean, indicating that these phytohormones could increase heat tolerance to sustain growth and yield (Khan et al., 2020).

Ethylene has a negative role in heat tolerance in plants (Rial et al., 2022). Earlier studies reported that heat stress leads to the increase in the production of ethylene contents along with oxidative damage, which lowers the pod setting percentage (Djanaguiraman et al., 2011). Abscisic acid (ABA) and GA are key hormones in regulating seed germination and dormancy. These hormones modify the growth and development of crops during heat stress (Manan and Zhao, 2020). Auxin was applied to soybean seeds which increased the germination percentage by increasing the biosynthesis of ABA (Shuai et al., 2017). However, hormonal content alterations and signaling in soybean seed germination under heat episodes remain unclear and need further studies (Sabagh et al., 2020). Ethylene (ET) is a main gaseous hormone that affects seed germination, growth and development, and fruit production under heat stress (Meena et al., 2020b). This hormone provides resistance to plants against abiotic stresses (Cao et al., 2007; Meena et al., 2020a). Ethylene triggers the expression of genes involved in heat tolerance and affects the osmolytes, which can protect plants under heat stress (Cui et al., 2015).

Further studies are needed to investigate the effect of ethylene on seed germination under adverse environmental conditions (Cui et al., 2015). Leaf senescence in soybean occurs during the reproductive stage and can be increased under heat stress. In a recent study, ethylene role in premature leaf senescence was investigated in soybean. The results identified that heat stress increased ethylene production in soybean leaves. These results showed the prominent role of ethylene in mitigating the adverse effects of heat stress in soybean (Djanaguiraman and Prasad, 2010). However, further studies must critically investigate ethylene role in inducing soybean heat tolerance (Djanaguiraman and Prasad, 2010).

**FIGURE 4:**
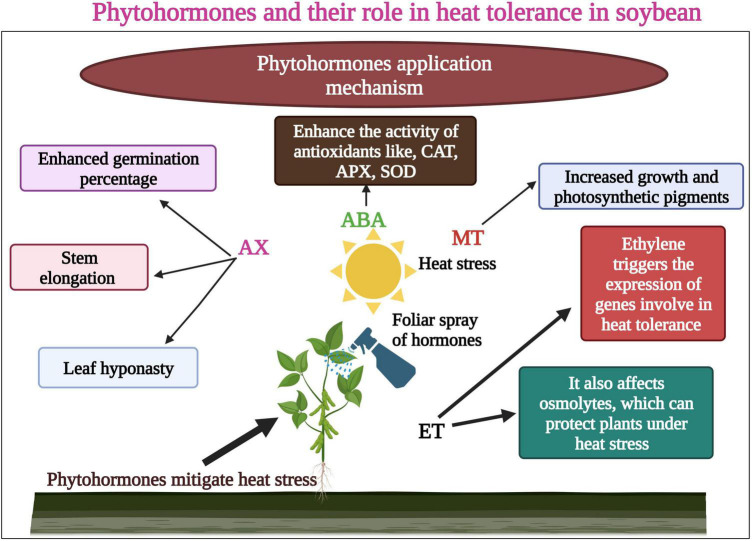
Role of phytohormones in heat tolerance in soybean. Hormones enhance seed germination, stem elongation, and enhanced photosynthetic pigments. Ethylene (ET) affects osmolytes which protect plants under heat stress. ET also enhances the function of genes involved in heat tolerance. This Figure is created with BioRender.com.

Melatonin (MT) is one of the most significant and vital osmoprotectants, is involved in numerous physiological processes in plants, and has a crucial role in triggering the antioxidant defense response in plants. Therefore, the uses of MT in plants are being assessed by many researchers. Wei et al. (2015) examined the effect of MT on the growth and development of soybean. Results revealed that MT application increased soybean seed size and plant height. Besides this, MT also enhanced the seed and pod numbers, but it did not increase the 100-seed weight under abiotic stresses (Wei et al., 2015). Likewise, MT use could increase the soybean tolerance to heat stress; the evidence shows that contrary environmental circumstances can increase the MT concentration in plants as a defensive response. In recent studies, Imran et al. (2021) revealed the role of MT a vital plant growth hormone, in reducing the harmful effects of heat waves on soybean. Heat stress produced ROS and disturbed plant growth and development. By foliar application of MT, plant growth and photosynthetic pigments (Figure 4) also increased in heat-stressed soybean seedlings.

Further, MT induced the expression of heat shock proteins and transcription factors, which indicated ROS detoxification (Imran et al., 2021). In another study, Kumar et al. (2021) revealed that MT promotes cell division, isoflavones, and ethylene biosynthesis in soybean under heat stress. The use of phytohormones to mitigate the toxic effects of heat stress in soybean needs further investigation (Kumar et al., 2021). Modified concentrations of hormones at different soybean growth stages under heat stress would help us sustain the soybean growth and yield in changing environments. The hormones and their interactions with the plant at different growth stages are critical to understanding. Further studies are required to investigate the potential role of SA in mitigating the adverse effects of heat stress in soybean.

## Other adaptive mechanisms

Under heat stress conditions, the plant adopts various mechanisms like short-term escape and avoidance, changed leaf orientation, membrane lipid functions, cooling, early maturity, and so on for survival (Shanmugavel et al., 2020). The plant also shows different degrees of leaf rolling under heat stress (Sarieva et al., 2010). Increased stomatal activities, a waxy layer, and enhanced xylem vessels are common survival tactics during heat stress. The plant also adopts a long-term tolerance mechanism to survive under heat stress (Srivastava et al., 2012). The use of antioxidants, late embryogenesis, and dehydrins are the main factors used to counter the harmful effects of heat stress. Osmolytes like proline, trehalose, and glycine betaine (GB) play a crucial role in osmotic adjustment, ROS scavenging, and membrane function stabilization under heat stress (Verbruggen and Hermans, 2008; Campobenedetto et al., 2020).

Numerous enzymatic and non-enzymatic antioxidant defense mechanisms are also involved in defense against oxidative stress brought by free radicals (Apel and Hirt, 2004). The action of enzymes involved in the scavenging ROS is temperature specific. Most of the antioxidant enzymes show improved activity with the rise in temperatures. It is also affected by the plant genotype, planting season, and phenological phases (Almeselmani et al., 2006). Campobenedetto et al. (2020) studied the role of lignin-derived biostimulants seed treatment in heat tolerance in soybean. They have observed a dramatic reduction in H_2_O_2_ activity associated with a substantial increase in non-protein thiols. These findings suggested the lower oxidative stress in biostimulants treated seed (Campobenedetto et al., 2020). These results offer perceptions of the biostimulants mechanism of action and its uses for seed treatment to enhance heat stress tolerance during seed germination in soybean (Campobenedetto et al., 2020). Nutrients also increase plants growth and yield under abiotic stress conditions (Saud et al., 2017) and increasing plant nutrition can lead to better adaptation under changing environmental conditions. (Ali et al., 2022a,b). The defense role of antioxidant enzymes needs to be improved to help plants withstand heat stress effects. Early maturing soybean should be the primary breeding target in future studies.

## Molecular techniques for heat tolerance in soybean

Genome-wide association study (GWAS) also assists in identifying traits-marker associations and knowing the genes controlling heat tolerance in soybean (Longmei et al., 2021). Using genomic studies and phenotypic data can provide information to the crop breeders to make a rapid selection and use advanced breeding tools to develop heat resilient crops. GWAS is one of the most promising tools to identify the QTL underlying particular traits of interest. The genetic control of heat stress analysis *via* association mapping would accelerate breeding programs (Kulwal et al., 2011). GWAS would identify the genomic regions governing the essential genes by conducting statistical links between traits variation and DNA polymorphism in extensive germplasm collection (Pandey et al., 2014). GWAS has increased the mapping resolution of genes/QTL in many crops (Ma et al., 2012; Liu et al., 2013). GWAS not only simplified to recognition of the marker-trait link but also advanced our knowledge of the genetic construction of complex quantitative characters (Yan et al., 2011). In response to heat stress, many stress-responsive proteins have been identified as heat shock proteins (HSP), indicating the plant tolerance threshold (Wang et al., 2004). Das et al. (2016) identified the 25 heat stress-related protein in soybean and revealed that EF-Tu triggered heat tolerance mechanisms. A higher level of HSPs regulated the heat tolerance mechanisms in soybean (Das et al., 2016). Katam et al. (2020) also studied protein’s response to soybean heat stress. These studies highlighted protein’s based effective heat tolerance mechanism (Katam et al., 2020). Proteins expression significantly changed within 3 h of heat stress in soybean roots. Proteins played significant role in heat tolerance (Valdés-López et al., 2016). Meanwhile, several other multi-omics techniques have great potential to enhance abiotic stress tolerance in crops, mainly using epigenomes in soybean (Chu et al., 2020). These techniques include genomics and microbiome (Liu et al., 2020; Pandian et al., 2020). Cao et al. (2022) presented a detailed review on use of multi-omics techniques for soybean molecular breeding. Machine learning tools play role in understanding the plants and environmental interactions. Hoffman et al. (2020) studied the soybean yield response to low temperature using Random Forest (RF), a diagnostic machine learning tool (Hoffman et al., 2020). Earlier, real time phenotyping framework using machine learning techniques was done in soybean to study the plant stress severity rating (Naik et al., 2017). Although the available data about these novel techniques for heat tolerance in soybean is insufficient, the critical information reported on other crops would assist in applying these powerful tools for developing heat tolerant soybean genotypes.

## Identification of quantitative trait loci for heat tolerance in soybean

Due to certain limitations of classical breeding, the development of heat-tolerant soybean genotypes is often limited. Conventional techniques are time-consuming, costly, and cannot fix the desired traits for a long time (Dhungana et al., 2021; Gillman et al., 2021). QTL mapping leads to identifying certain novel QTL controlling heat tolerance in soybean. Climate change may result in prolonged heat stress during germination and growth (Zhang et al., 2022b). The ancestors of modern US high-yielding soybean lack potential resistance to heat stress; however, some plant cultivars can maintain 95% yield and quality under extreme heat stress (Gillman et al., 2021). Gillman et al. (2021) evaluated the backcross recombinant inbred line (BRILs) population for seed emergence and germination produced in six greenhouses. They have found a novel QTL derived from “PI587982A” correlated with significant effects on overall seed germination and emergence. These results revealed that marker-assisted selection (MAS) could be practiced to increase tolerance to heat stress and may lead to the development of cultivars with high-quality seeds in soybean (Gillman et al., 2021). Using a stable mapping population and wild relatives of soybean would be fruitful in identifying novel QTL and potential genes network behind heat tolerance and developing heat-tolerant cultivars (Gillman et al., 2021). The information about QTL mapping for heat tolerance in soybean is limited, strongly emphasizing further studies using diverse mapping populations to identify the genomic regions controlling heat tolerance in soybean (Gillman et al., 2021). The process needs evaluation of soybean in different temperature regimes to validate the potent QTL and use them in MAS to develop heat-tolerant soybean genotypes.

## Genetic engineering for heat tolerance in soybean

Genetic engineering leads to the development of several heat-tolerant soybean genotypes (Arce-Paredes et al., 2011). Transgenic soybean expressed transgene in high-temperature regimes and showed a high yield compared to control genotypes. Heat shock proteins are an essential component of plant response to heat stress. Earlier, Huang et al. (2019) investigated the role of the *GmHsp90A2* gene in soybean using transgenic lines generated by CRISPR/Cas9 system. *Agrobacterium tumefaciens* strain EHA105 was used for plant transformation. The T_3_ transgenic soybean plants with overexpression of *GmHsp90A2* exhibited heat tolerance through lower oxidative stress and high chlorophyll contents.

On the other hand, T_2_ transgenic lines showed high oxidative stress and low chlorophyll contents (Huang et al., 2019). Likewise, in another study, 75 non-redundant AQP encoding genes were recognized in soybean. Results revealed that the AQP gene- *GmTIP2;6* was involved in heat tolerance in soybean. This gene enhanced plant growth during heat stress, and these results provided the way to understand the molecular mechanism of *GmAQPs* in mediating heat stress (Feng et al., 2019). The ortholog of soybean *GmGBP1* positively regulated the heat tolerance not only in soybean but also in tobacco. This gene enhanced seed germination during heat stress and contributed to thermal tolerance in soybean (Zhao et al., 2013).

**FIGURE 5:**
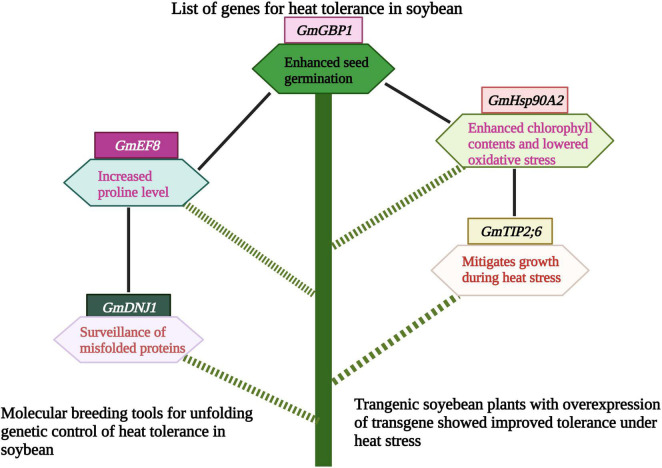
Role of different genes/proteins in heat tolerance in soybean. Heat tolerance genes protect soybean plants by various mechanisms like, increasing proline level, mitigating growth and photosynthesis. This Figure is created with BioRender.com.

Soybean family protein, *GmEF8* enhanced heat tolerance by increasing the proline level in transgenic lines. These results revealed the potential role of the *GmEF8* (Figure 5 and Table 3) gene under heat stress. They provided a foundation for further studies to understand the complexities of stress transduction pathways (Zhang et al., 2022a). The soybean gene, *Gmdnj1* positively regulated the heat tolerance in transgenic lines. Results showed that *Gmdnj1* enhanced heat tolerance by searching misfolded proteins for refolding to sustain the total capacity of cellular roles (Li et al., 2021). Nowadays, transgenic techniques are practiced using efficient transformation vectors. However, available data is insufficient to comprehensively evaluate transgenic lines under extreme heat stress conditions. It would be better to generate a substantial germplasm center and identify the gene for heat tolerance and transfer using *Agrobacterium* vectors (Li et al., 2021). Transgenic soybean performed best under extreme heat stress conditions; however, further studies are required to identify more potent genes for genetic engineering to develop heat-tolerant genotypes.

**TABLE 3:** Genetic engineering for heat tolerance in soybean.

Crop	Gene/Protein	Transformation vector	Function	References
Soybean	*GmGBP1*	*Agrobacterium tumefaciens* LBA4404	Enhanced seed germination	Zhao et al., 2013
	*GmHsp90A2*	*Agrobacterium tumefaciens* strain EHA105	Enhanced chlorophyll contents and lowered oxidative stress	Huang et al., 2019
	*GmTIP2;6*	*Agrobacterium tumefaciens strain* GV3101	Mitigates growth during heat stress	Feng et al., 2019
	*GmDNJ1*	*Agrobacterium tumefaciens strain* LBA4404	Surveillance of misfolded proteins	Li et al., 2021
	*GmEF8*	*Agrobacterium tumefaciens*	Increased proline level	Zhang et al., 2022a

## CRISPR/Cas9 role in heat tolerance

Soybean tolerance to heat stress is often limited because of the complexity of its genome. With time, the emergence of novel gene-editing tools like CRISPR/Cas9 broke all biological barriers (Ghosh and Dey, 2022; Rasheed et al., 2022a; Razzaq et al., 2022). Increased heat waves threaten food security and damage the soybean crop on large areas (Ahmad et al., 2021; Chennakesavulu et al., 2021; Camerlengo et al., 2022; Pandita, 2022). CRISPR/Ca9 has emerged as a key gene-editing tool for efficient and precise gene-editing of soybean crop; however, little evidence indicates its role in heat tolerance. There are possibilities of large-scale use of this tool in future research studies. Secondly, CRISPR/Cas9 variants would expand its role in increasing soybean tolerance to not only heat stress but also other abiotic stresses. Earlier, CRISPR/Cas9 successfully edited the heat tolerant genes in soybean (Huang et al., 2019). CRISPR/Cas9 edited two genes, *GmpPLA-II*ε and *GmpPLA-II*ζ, in response to multiple abiotic stresses. These findings revealed that these genes could also play a key role in heat stress tolerance in soybean. The mutants would be valuable genetic materials to investigate the molecular mechanism governing soybean response to multiple abiotic stresses (Xiao et al., 2021) and hopefully for heat tolerance. Yang et al. (2022) also applied CRISPR/Cas9 to edit the heat shock protein, *GmHSP17.9* The resulting mutant plants showed a remarkable variation in nodule number, nodule fresh weight, and nitrogenase activity. These findings reveal an unprecedented molecular mechanism of heat shock protein in nodule development and counter heat stress in soybean. Hopefully, use of CRISPR/Cas9 will increase in soybean to develop heat resilient cultivars. Complete genome sequencing has been completed in soybean, which will be helpful in the future to screen for heat tolerant genes that can be targeted by CRISPR/Cas9.

## Transcription factors for heat tolerance in soybean

Transcription factors are proteins that regulate gene expression in soybean (Liu et al., 2022a,b; Min et al., 2022; Yin et al., 2022). Hundreds of TFs have been identified and characterized for heat tolerance in soybean. To respond to heat waves, plants activate heat shock proteins characterized by their highly conserved modular structure and motif. In an earlier study, a potent heat shock TF, *GmHsfA1*, was identified and distinguished from expressed sequence tag database of soybean by sequence comparison with *LpHsfA1* and cloning of complementary DNA (cDNA). Soybean transgenic plants overexpressed *GmHsfA1*, leading to the activation of *GmHsp70* under control conditions and overexpression under high temperatures. The overexpression of *GmHsfA1* in transgenic plants enhanced thermotolerance (Zhu et al., 2006).

In the same way, in another study, a novel *GmHSFA1*was cloned from the soybean genome. Results showed that expression of *GmHSFA1* was higher in transgenic lines than in non-transgenic soybean. *GmHSFA1* increased thermotolerance in transgenic soybean by activating several genes (Chen et al., 2006). Several abiotic factors restrict the soybean yield, and no soybean genotype with improved tolerance to heat stress is commercially available.

Ribichich et al. (2020) transformed the sunflower TF, *HaHB4* into soybean, which confers drought tolerance in *Arabidopsis*. Results showed that transgenic plants showed greater heat tolerance in field conditions. It was assumed that this TF, *HaHB4*, could control soybean yield under warm conditions (Ribichich et al., 2020). Soybean TF, *DREB1*, plays a crucial role in activating gene expression in response to heat stress. 14 DREB1-type TFs were identified from the soybean genome database. The expression of *GmDREB1* was enhanced by heat stress. Results suggested that *GmDREB1* proteins activate the heat-responsive genes in soybean and increase heat tolerance (Kidokoro et al., 2015). Likewise, heat shock TF *GmHsf*s also activates heat-responsive genes in soybean. This TF regulates heat shock protein expression in response to extreme heat waves in soybean (Chung et al., 2013). Li et al. (2021) identified a type-I HSP40 gene *GmDNJ1* in soybean, which is highly inducible under heat stress. Mutants of *Gmdnj1* exhibited reduced chlorophyll contents, oxidative stress, and higher induction of heat-responsive genes. *GmDNJ1* plays a key role in plant growth and development under heat stress *via* the regulation of misfolded proteins (Li et al., 2021). Transcription factor *BREB2* in soybean enhanced heat tolerance responses by activating several genes. The regulatory mechanism of *DREB2* in soybean is not clear. The novel DREB2 gene, *GmDREB2A;2*, was highly induced under heat stress (Table 4). This gene has a key role in thermotolerance in soybean and can be used as a candidate gene for future studies (Mizoi et al., 2013).

**TABLE 4:** List of several heat-responsive TFs in soybean.

Transcription factors (TFs), genes, proteins	Function	References
*GmDNJ1*	Higher induction of heat-responsive genes, sugar metabolism, and membrane transport	Li et al., 2021
*HaHB4*	Enhanced yield, greater epicotyl diameter, and larger xylem area	Ribichich et al., 2020
HSPs	Unregulated and expressed in prolonged heat stress conditions	Song et al., 2017
*GmDREB1*	Activates the *GmPYL21* gene to enhance heat tolerance	Kidokoro et al., 2015
*GmHsf-34*	Overexpressed and improved tolerance to heat stress	Li et al., 2014
*GmHsfs*	Activates the heat shock proteins	Chung et al., 2013
*GmHsfA1*	Enhanced heat tolerance by activation of genes	Zhu et al., 2006
DREB2/*GmDREB2A;2*	Activates stress-responsive gene	Mizoi et al., 2013
*GmHSFA1*	Mediated the activation of transcription	Chen et al., 2006

In soybean, *GmEF8* is an elongation factor family protein determined by interaction with *GmCBL1*, and *GmEF8* expression was induced by heat stress. Soybean plants with enhanced tolerance to heat stress showed overexpression of *GmEF8* in hairy roots and had a high proline level compared to plants with control hairy roots (Zhang et al., 2022a). Earlier, Song et al. (2017) performed an RNA-seq analysis using two soybean varieties under extreme stress. They analyzed the transcripts and obtained 2,458 differentially expressed genes. In the end, 68 genes were found involved in heat shock proteins under heat stress. As a result, small HSP families were found to be upregulated and expressed in prolonged heat stress conditions. These findings provided valuable information about heat tolerance in soybean (Song et al., 2017). Heat shock TFs play a key role in helping soybean plants in extreme heat stress episodes. In another study, thirty-eight Hsfs were analytically recognized, assembled into three classes and 12 subclasses, and positioned on 15 soybean chromosomes. A1 and A2 subclass showed a high transcription level. Numerous genes were selected for their subcellular localization. Results of subcellular localization showed that *GmHsf-04* and *GmHsf-34* were positioned in the nucleus. *GmHsf-*34 gene overexpressed and improved soybean tolerance to heat stresses (Li et al., 2014). Despite the significant progress in enhancing heat tolerance by identifying TFs, the actual and core mechanism of TFs under heat stress is not fully understood. It would be better to expose soybean plants to extreme heat episodes and identify the potential TFs involved in heat tolerance. It would be better to fully characterize the soybean genome (Aleem et al., 2022; Goodman and Kiser, 2022), arrange the gene into groups based on their expression in a cell under abiotic stress and select the genes with high overexpression (Zhang et al., 2022c). Different TFs families, WRKY, bZIP, and NAC should be studied to identify the family members involved in heat tolerance. TFs could be a potential target for genetic engineering and CRISPR/Cas9.

## Transcriptome analysis for heat tolerance in soybean

Transcriptome analysis plays a crucial role in identifying stress-responsive genes in soybean (Sun et al., 2021; Chen et al., 2022; Ma et al., 2022; Tariq et al., 2022). Soybean has a complex genetic mechanism for heat tolerance. Knowledge about soybean genetic mechanism of heat tolerance is critical for molecular breeding (Yang et al., 2022). To identify the genetic mechanism of heat stress and open ways for molecular breeding, researchers have conducted several studies to identify the potential gene involvement in heat tolerance in soybean. Wang et al. (2018) used RNA-sequencing to investigate the transcriptional response of soybean toward heat stress. Several genes involved in defense, metabolic pathways, photosynthesis, and morphological responses are expressed differently under heat waves. Results showed 1,468 and 1,220 upregulated and 1,146 and 686 down-regulated genes after heat treatment (8 h and 24 h). These genes are actively involved in defense response. These genes improved heat tolerance in soybean *via* molecular breeding (Wang et al., 2018). Likewise, in another study, transcriptome analysis was performed to investigate the role of lignin-derived biostimulants seed treatment in enhancing heat tolerance in soybean. RNA-Sequence analysis showed 51 upregulated genes and 828 down-regulated genes in biostimulants treated seed after 24 h of treatment compared to control. Down-regulated genes were involved in heat stress-responsive and hormonal signaling. Genes activation under heat stress improves heat tolerance *via* molecular breeding (Campobenedetto et al., 2020).

Soybean roots play a crucial role in developing the symbiotic association with microorganisms, but unfortunately, few studies described their possible response to heat stress. Genome-wide transcriptome and proteomic analyses were performed on isolated soybean roots to investigate their response to heat stress. On average, 1,849 and 3,091 genes that were differentially expressed under heat stress were identified. Only ten essential genes were involved in the heat-responsive mechanism in soybean. Besides this, a proteomic analysis was also performed on membrane fractions detached from root hairs. Proteomic analysis identified proteins whose expression was changed after heat stress treatment for 3 h. Most of these identified proteins were actively involved in heat tolerance in soybean (Valdés-López et al., 2016). High-temperature stress affects seed development and maturity and leads to seed deterioration; however, protein involvement is not fully understood. Wang et al. (2012) compared the proteome composition of developing seed, and 42 proteins were identified and belonged to 31 diverse protein species. These proteins involved in 13 cellular response pathways, including protein folding, protein biosynthesis, and carbohydrate metabolism, etc. Such a mechanism allows us to investigate the management techniques to maintain soybean growth under heat stress (Wang et al., 2012).

Many studies reported the role of transcriptomes and proteins in drought stress response in soybean; however, studies about heat tolerance are limited. We emphasize conducting more studies to identify the potential genes governing the heat-responsive pathways in soybean. Exposing plants to varied heat stress levels would be better in analyzing transcriptional gene profiles and investigating their regulatory network. Likewise, the proteomic analysis would show the active proteins in soybean heat tolerance. With the advancement in the molecular field and the discovery of new tools, the speed of molecular breeding would increase and allow us to sustain crop growth under changing environmental conditions.

## Conclusions and future research directions

Climatic changes are continuously threatening global food security. Industrialization leads to increased heat waves because of the global warming threats. Heat waves decreased the yield of the crop to a significant level. The continuous expansion of the human population threatens our ability to feed the entire world. Scientists have increased crops yield under changing climatic conditions. Soybean is a significant oilseed crop and part of the human diet and serves as a primary protein source of livestock feed. Crop responses to heat stress involve morphological, physiological, and molecular pathways. In conventional ways, plants use avoidance, escape, and tolerance mechanisms to cope with heat episodes. The difference in protein contents also contributes to heat tolerance in soybean. Variation in stomatal closure and leaf angle is linked with the morpho-physiological type of response. Certain hormones like CK, MT, and ET can help soybean plants counter the harmful effects of heat waves in maintaining yield and quality. The role of other phytohormones is not yet fully understood and needs further studies. This is one of the most potent agronomic approaches to compensate for yield loss in soybean under heat stress conditions. High-yielding soybean varieties have been developed to address food security challenges, and breeders are engaged in bringing genetic variation for heat tolerance in soybean. Conventional breeding methods are time-consuming and costly, so they have limited use in modern agriculture and can be modified for better results.

There are specific challenges in improving heat stress tolerance in soybean. These challenges include lack of complete genetic information, insufficient knowledge about crop nature, genetic erosion, lack of research on wild soybean types for gene identification, and limited use of the latest molecular techniques for heat tolerance. Heat tolerance is a polygenic trait and cannot be fully developed by traditional breeding methods. Hence, molecular techniques like QTL mapping, genetic engineering, CRISPR/Cas9, transcriptome sequencing, and transcription factors identification are reliable genetic approaches to develop heat-tolerant soybean genotypes. Many success stories have been published, indicating the reliability of these methods in understanding the genetic mechanism of heat tolerance in soybean. We recommend the CRISPR/Cas9 as the most promising and reliable tool for targeted editing of heat tolerance genes in soybean, as it can works across all biological boundaries. Unfortunately, QTL mapping for heat tolerance in soybean is limited, and only a few reports have been published. QTL mapping area must be broadened to identify the potential regions involved in heat tolerance in soybean. This review emphasizes using wild relatives to increase genetic diversity for soybean heat tolerance in the future. Transcription and transcriptome analysis played a key role in heat tolerance in soybean; however, there is a big gap in these studies that should be filled. Complete genome sequencing will lead to the detailed characterization of all heat-tolerant genes and their possible use in future breeding plans. By adopting these techniques and combining efforts, soybean production can be maintained under heat stress conditions, leading to a green revolution.

## Author contributions

GJ, GY, GuY, and AR prepared the manuscript. ZQ and XZ prepared the figures. AM, ZS, ZoZ, ZnZ, WX, and WJ involved in reviewing and editing. All authors contributed to the article and approved the submitted version.
